# Pharmacological Characterization and Raman Spectroscopy Evaluation of Oral and Maxillofacial Surgery-Related Carnoy´S Solution Modified by Different Viscosity Agents

**DOI:** 10.31557/APJCP.2019.20.11.3335

**Published:** 2019

**Authors:** Francisco Samuel Rodrigues Carvalho, Marina Mota Lima Verde, Khalil Fernandes Viana, Thâmara Manoela Marinho Bezerra, Said Gonçalves da Cruz Fonseca, Karuza Maria Alves Pereira, Thyciana Rodrigues Ribeiro, Fábio Wildson Gurgel Costa

**Affiliations:** 1 *Division of Oral and Maxillofacial Surgery, Federal University of Ceará Campus Sobral, Sobral, *; 2 *Dentist, UNIFOR, *; 3 *Division of Oral Pathology, *; 4 *Division of Oral Pathology,*; 5 *Division of Pharmacology Science, *; 6 *Department of Morphology, School of Medicine,*; 7 *Division of Patients with Special Needs, *; 8 *Division of Oral Radiology, Walter Cantídio University Hospital, Federal University of Ceará, Fortaleza, Brazil. *

**Keywords:** Carnoy’s solution, oral surgery, Raman Spectroscopy

## Abstract

**Background:**

There are several lesions of odontogenic and non-odontogenic origin in the oral cavity, such as odontogenic keratocyst, as well as many treatment options for such lesions. In order to reduce recurrence due to conservative treatments and less aesthetic and functional impairment of the patient (radical therapies), Carnoy’s solution has been used as an adjuvant to surgery, showing satisfactory results. Its application is not standardized, presenting risks to adjacent tissues. Thus, we characterized the Carnoy’s solution with different viscosity agents to enhance its applicability.

**Material and Methods:**

All solutions prepared (Carnoy with and without chloroform) were added with viscosity agent: ethyl cellulose, propylene glycol, and glycerol totaling eight solutions. The pharmacological characterization of the solutions was performed by determining the mass density and relative density (using a clean and dry pycnometer), pH (using pH meter), and concentration of Fe^3+^ (using ultraviolet/visible spectroscopy). The analyses of the inorganic components were determined by Raman micro spectrometry. Data were analyzed with statistical program BIOESTAT 5.3.

**Results:**

Solutions with ethyl cellulose were discarded due to precipitate formation and suspension of the viscosity agent. In the other solutions, viscosity increase (propylene glycol solutions) and acidic pH were observed mainly in the glycerol group. The ferric chloride characterized as a hemostatic agent had its concentration increased with the use of thickening agents, theoretically favoring its action.

**Conclusion:**

The similarity of the propylene glycol and glycerol molecules justifies the Raman spectra of these substances to be similar and the difficulty in obtaining a “fingerprint”.

## Introduction

The dental surgeon may find in his clinical practice several lesions, odontogenic and non-odontogenic, affecting the maxillofacial complex (Cavalcante et al., 2016; da Silva et al., 2018). Some of these lesions are not necessarily associated with painful symptoms and are diagnosed on routine imaging tests (Alves et al., 2018). Among these lesions we can highlight odontogenic keratocyst (OKT) and amelobastoma, which, despite being a benign pathology, is locally invasive and associated with high rates of relapse. Conservative treatment may be associated with recurrence, while radical surgery may result in aesthetic-functional impairment (Alves et al., 2018).

Several modalities of therapy complementary to surgery have been widely used, among them cryotherapy and the use of the Carnoy’s solution (Stoelinga, 2001; Stoelinga and Bronkhorst, 1988; Voorsmit, 1985; Voorsmit et al., 1981). A systematic review with a meta-analysis of 2.287 odontogenic tumors observed lower rates and a more extended period of time for recurrence when the treatment modality consisted of enucleation using Carnoy’s solution (11.5%) or surgical resection (8.4%) (Al-Moraissi et al., 2017). Complications and morbidity from the treatment with Carnoy’s solution have a lower frequency and degree of severity when compared to surgical resection (Alchalabi et al., 2017). 

Initially formulated for botanical studies, Carnoy’s solution contains 6 ml of absolute alcohol, 3 ml of chloroform, and 1 ml of glacial acetic acid. The first modification of the solution was the addition of 1 g of ferric chloride to provide hemostatic characteristics. Used as a complementary therapy to surgery, the Carnoy’s solution was applied in patients only in 1933, for the treatment of cysts and cutaneous fistulas. The pioneers in the use of the solution for mandibular lesions were Voorsmit and Stoelinga, in 1981, for treatment of OKTs (Alves et al., 2018). Its use promotes superficial necrosis of the tissue, from a chemical cauterization up to 1.5mm deep after 5 minutes of application in bone stores (Blanas et al., 2000; Carvalho et al., 2018; Voorsmit, 1985). 

Surgeons associate the Carnoy’s solution acts as a corrosive agent shallow penetration that functions as adjuvant conservative therapy in the treatment of odontogenic tumors such as OKT (Al Moraissi et al, 2016; Alchalabi et al, 2017; Alves et al., 2018), amelobastoma (Lee et al., 2012; Alves et al., 2018), while pathologists recognize the solution as a fixative for microscopic analysis (Dias et al., 2016).

The second modification of the solution was due to a 1992 Food and Drug Administration (FDA) ruling, which vetoed the use of chloroform for therapeutic purposes (Carvalho et al., 2018). Thus, the 3ml of chloroform was replaced by the addition of 3ml of absolute alcohol, totaling 9 ml in solution. This substitution was done due to the possible damages that this chemical could cause to the health since it is considered possibly carcinogenic (Carvalho et al., 2018). However, results found in some studies, support the relevance of chloroform maintenance in the solution, due to the lower rates of recurrence and better performance of the solution compared to the solution modified by the removal of chloroform (Carvalho et al., 2018).

The application of Carnoy’s solution in the tissues does not have standardization. It is applied on the lesion surface before its enucleation (Voorsmit et al., 1981) or on the bone tissue after the enucleation (Carvalho et al., 2018; Leung et al., 2016). Also, it can be injected in the soft tissues adjacent to a lesion or recoating mucosa (Al-Moraissi et al., 2017; Al-Moraissi et al., 2016; Al-Moraissi et al., 2016; Stoelinga, 2005). The use of solution presents a risk of mucosal lesion, gingival edema, nerve damage, among others (Alchalabi et al., 2017). One of the ways to control and ensure homogenous application and release of a topical drug is the use of viscosity polymers. These can be associated with the most variable pharmaceutical formulas, without toxic effect, to form release matrices or even membranes containing the active principle, guaranteeing greater control in its use in the applied place (Pillai and Panchagnula, 2001; Yadav et al., 2017). 

We made a modified Carnoy solution with the addition of different viscosity agents, aiming to improve the applicability of the solution to bone surfaces. Through the analysis Raman spectroscopy, we obtained spectral patterns of each substance allowing its specific identification in the middle of a compound, giving us the “fingerprint” of each substrate. Raman spectroscopy promotes the passage of a molecule from its natural state to a vibrational state, in this way, since each molecular specimen has its propper vibration pattern, it is possible to identify each molecule studied (Kann et al., 2015; Kong et al., 2015).

In this context, the aim of the present study was to evaluate the alterations in Carnoy’s solution formulations with different viscosity agents and compare them with conventional formulations with and without chloroform.

## Materials and Methods

*Obtaining the solutions*

The Carnoy solutions containing chloroform were prepared following the same protocol described by Carvalho et al., (2018). For the preparation of the Carnoy solution without chloroform, 3 mL of chloroform was replaced by the addition of 3 mL of absolute alcohol. Viscosity change occurred by insertion of ethyl cellulose (5ml), glycerol (5ml), or propylene glycol (5ml), materials selected for use in oral cavity gels. Thus, there were eight solutions to be analyzed.

*Pharmacological characterization (density, pH, viscosity, Fe* ^3+^ *concentration)*

Pharmacological characterization of the solutions was performed by determining the mass density and relative density using a clean and dry pycnometer (Prolab Picnometer Materials of laboratory, São Paulo - SP), with a capacity of 12.7 ml previously calibrated and Ostwald viscometer (Viscotester 6L Viscometer Thermo Haake - Thermo Fisher Scientif Inc, Germany). A pH meter was used to determine the pH (bench pH meter model DM 23 - Digimed, Campo Grande Brazil). The concentration of Fe^3+^ was determined using ultraviolet / visible (UV / visible) spectroscopy (GENESYS 20 UV/ VIS Spectrophotometer, Thermo Fisher Scientific Inc., Germany), by reacting potassium thiocyanate in aqueous solution (Tang et al., 2016).

*Raman spectroscopy*

The spatial distribution of the inorganic components was determined by the relative intensities corresponding to the peaks of Raman micro spectrometry (Xplora, Horiba Scientific, Paris, France). For this purpose, an argon laser with a wavelength of 638 nm and a power of 3.2 mW (Olympus American Inc) was used. In addition, the analyses were performed at a 10x magnification where the focus was performed on the surface of the solution that was in a glass cuvette. The Raman spectra were obtained in the range of 200 cm^-1^ to 3,500 cm^-1^ with three accumulations of 10 seconds. 

All the readings were performed at the same temperature and pressure (22ºC and 1 atm), using 10ml for analysis (Carvalho et al., 2018). Before data acquisition, the apparatus was calibrated with silica with a wavelength of 520 cm^-1^, as recommended by the manufacturer. Subsequently, all spectra were analyzed and manipulated using OriginPro 9.0 software (OriginLab Corporation, Northampton, Massachusetts, USA). All data were processed with baseline correction, smoothing by the polynomial method (Savitzk-Golay), and the peaks were identified as to their position and intensity through the Gaussian and Lorentzian method to determine the characterization and deconvolution of the graphs (Nouri et al., 2015).

*Data analysis*

All statistical analysis was performed using software Bioestat 5.3. Statistical analysis of the different viscosity agents and concentration of [Fe^3+^] in Carnoy’s solution without chloroform were performed by unpaired Student’s t-test. The significance level of 5% was adopted, and the analyses were performed through the software Bioestat 5.3. 

## Results

*Pharmacological Characterization*

Carnoy’s solutions with and without chloroform, when mixed with ethyl cellulose (5ml), showed precipitation of material at the bottom of the flask and presence of undiluted thickening agent in solution. Thus, this study group was removed.

The relative density and viscosity of Carnoy’s solution were influenced by the presence of chloroform and viscosity agents. When present, viscosity agents render chloroform-free solutions more viscous, unlike that found in Carnoy’s solutions without viscosity agents. Despite this characteristic, the viscosity was increased in all groups. 

The pH of all solutions is highly acidic, and these are influenced by the presence of chloroform. The presence of the viscosity agent appears to reduce the difference in pH increase when compared to Carnoy’s solution with and without chloroform plus different viscosity agents. The pH had higher acidity when glycerol was used in comparison to propylene glycol, despite the decrease in viscosity (Table 1).

The concentration of Fe^3+^ ions analyzed by UV / Visible spectroscopy showed a slight increase in solutions without chloroform when the viscosity agents were used. While in the presence of chloroform it presented lower values (Tables 2 and 3).

*Raman spectroscopy*

**Figure 1:**
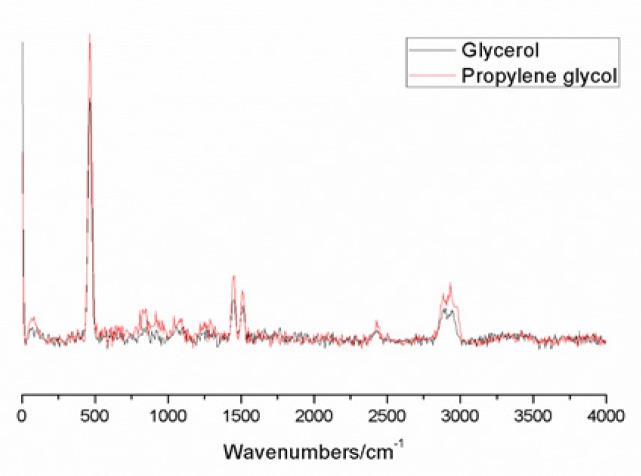
Spectra of Glycerol and Propylene Glycol with a Red Laser (638nm) Compiled in OriginPro 9.0 Software®.

The spectra of the viscosity agents (Figure 1) were obtained and described for the relative intensity of the peaks in relation to the highest peak of the substance, determining from there the peaks in very weak, weak, medium, strong, very strong and shoulders (Table 4).

**Figure 2:**
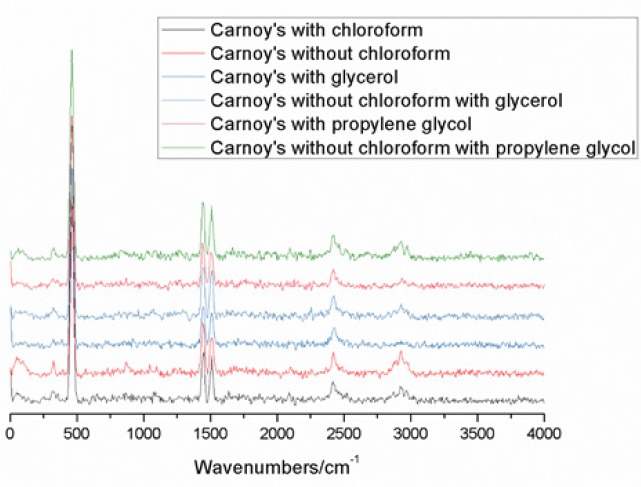
Carnoy’s Solution with and without Chloroform and Viscosity agents Evidencing the most Significant Variation between 2,500 cm^-1^ and 3,500 cm^-1^, with Red Laser (638 nm) Compiled in OriginPro 9.0 Software^®^.

The spectra of the Carnoy solutions were superimposed on graphs that were quite similar. The region between 2,500 cm^-1^ and 3,500 cm^-1^ was the one with the greatest variation since the variations between the peaks in the other regions of the analyzed spectrum were small (Figure 2).

**Table 1:** Pharmacological Characterization of Carnoy’s Solution with and without Chloroform Associated with Differents Viscosity agents in Relation to Water

	Mean spreading time (s)	Density (g/ml)	Viscosity (mPa*s)	pH
Water	7.24	0.99704	1	7.0
Carnoy’s solution with chloroform	7.36	10.805	1.1017^A^	0.12ª
Carnoy’s solution with chloroform + glycerol	7.66	11.049	1.1725^A^	0.04^a^
Carnoy’s solution with chloroform + propylene glycol	7.94	11.289	1.2417^A^	0.22^a^
Carnoy’s solution without chloroform	7.72	0.8862	0.9478^B^	0.56^b.c^
Carnoy’s solution without chloroform + glycerol	10.48	0.9861	1.4309^C^	0.09^d^
Carnoy’s solution without chloroform + propylene glycol	16.8	10.758	2.5037^D^	0.24^d^

Capital letters show statistical analysis for viscosity values, lowercase letters for pH values. Different letters correspond to the presence of statistical difference, while equal letters correspond to absence of statistical difference. A, p,0.3023; B, C, D,p<0.0001; a, p,0.1677; b, c, d, p,0.0032.

**Table 2:** The Concentration of [Fe^3+^] in Carnoy’s Solution with Chloroform by UV/Vis Calculated by the Line equation in the Calibration Curve (Abs = 0.1521.[Fe^3+^] + 0.0077)

Solution	[Fe^3+^] (μg/ml)
Carnoy’s solution	8.16
Carnoy’s solution + Glycerol	8.37
Carnoy’s solution + Propylene glycol	8.33

**Table 3:** The Concentration of [Fe^3+^] in Carnoy’s Solution without Chloroform by UV/Vis Calculated by the Line Equation in the Calibration Curve (Abs = 0.1521. [Fe^3+^] + 0.0077)

Solution	[Fe^3+^] (μg/ml)
Carnoy’s solution without chloroform	8.72^a^
Carnoy’s solution without chloroform + Glycerol	9.95^b^
Carnoy’s solution without chloroform + Propylene glycol	8.93^a^

Different letters correspond to the presence of statistical difference (p<0.0001).

**Table 4:** Characteristic Peaks of Viscosity Agents

Glycerol		Propylene glycol	
Peak	Intensity	Peak	Intensity
464	m	465	s
837	w	518	vw
914	vw	837	s
1052	w	914	m
1250	vw	1078	m
1454	w	1238	vw
2888	s	1280	vw
2941	s	1449	s
		1502	vw
		2878	vs
		2932	vs
		2972	vs

vw, very weak; w, weak; m, medium; s, strong; vs, very strong Underlined items can differentiate substances.

## Discussion

Ethyl cellulose did not prove to be a usable viscosity agent since the formulated solutions changed by precipitate formation and suspension of the thickening agent. The increase in viscosity was observed with the use of thickening agents. Propylene glycol showed a more significant increase over glycerol. Both agents had higher viscosity increase when associated with Carnoy’s solution without chloroform. This fact may ensure better clinical application of solutions with increased viscosity, whereas controlling the area of application of adjuvant therapy ensures a greater reduction of complications associated with the use of Carnoy’s solution (Ribeiro Júnior et al., 2012).

The pH of the solutions showed an acid pattern, the glycerol formulation being the one with the lowest values. These values can be explained by the presence of acetic acid, chloroform and ferric chloride in solution (Carvalho et al., 2018). Carnoy’s solution with chloroform was more clinically effective when compared to chloroform-free solution because the recurrence rates in OKT were lower in the first group (Dashow et al., 2015). Chloroform appears to be associated with a reduction in the pH of the solution and with the capacity of recurrence of the injuries (Carvalho et al., 2018). Thus, the authors believe that the use of glycerol and propylene glycol may show improvement in clinical outcomes when used since the pH is diminished in its use.

The concentration of Fe^3+^ appears to increase with the use of viscosity agents, being higher in the glycerol group than the propylene glycol group. It was noteworthy that the solutions that did not present chloroform in their composition obtained higher concentrations in relation to the solutions that presented chloroform.

Ferric chloride was characterized as a hemostatic agent that acts by coagulative necrosis, (Nouri et al., 2015), which from the therapeutic point of view may be associated with lower rates of relapse in odontogenic neoplasms (Carvalho et al., 2018). Thus, the use of viscosity agents may favor this characteristic since we observed an increase in Fe^3+^ ion concentration, mainly in glycerol.

The spectra of Carnoy’s solution can be differentiated for the presence or absence of chloroform, mainly in the region of 200 cm^-1^ at 1,600 cm^-1^. However, the use of viscosity agents did not allow the differentiation of peaks in the area. The main difference in the present study was observed in the region between 2,500 cm^-1^ and 3,500 cm^-1^, characterized mainly by the phenol and alcohol groups. These groups were present in all solutions since the solvent of the solutions was the alcohol, and the viscosity agents have such groups in their composition. Glycerol (C_3_H_8_O_3_) differs from propylene glycol (C_3_H_8_O_2_) by the presence of an oxygen molecule that has the most reason to justify the similarity of the Raman spectra of these substances. Therefore, obtaining a fingerprint for the solutions of the present study becomes challenging, since the peaks that are unique for a particular substance have low intensity.

In conclusions, Glycerol and propylene glycol were shown as possible viscosity agents for use with Carnoy’s solution with and without chloroform. Propylene glycol is associated with higher viscosity, and glycerol is associated with acidic pH and higher concentrations of Fe^3+^ ions in solution. The characterization of Carnoy’s solution with these viscosity agents can be hampered by the chemical similarity of the compounds and the alcohol component of the solutions.
